# Evaluation of Platelet Parameters in Patients with Pulmonary Hydatid Cyst

**DOI:** 10.4084/MJHID.2010.006

**Published:** 2010-04-14

**Authors:** Abdulkadir Küçükbayrak, Gürhan Öz, Göktürk Fındık, Nurettin Karaoğlanoğlu, Sadi Kaya, İrfan Taştepe, Engin Şenel, Zeynep Seçkin Küçükbayrak

**Affiliations:** 1 MD, Clinic of Infectious Diseases and Clinical Microbiology, Ataturk Chest Diseases and Thoracic Surgery Training and Research Hospital, Ankara, Turkey; 2 MD, Clinic of Thoracic Surgery, Ataturk Chest Diseases and Thoracic Surgery Training and Research Hospital, Ankara, Turkey; 3 MD, Clinic of Dermatology, Çankiri State Hospital, Çankiri, Turkey; 4 MD, Department of Physiology, Duzce University School of Medicine, Duzce, Turkey

## Abstract

**Background:**

*Echinococcosis* is a near-cosmopolitan zoonosis caused by adult or larval stages of tapeworms (cestodes) into the genus *Echinococcus* (family Taeniidae). It was demonstrated that platelets were capable of killing parasites independent from leukocytes. Purpose: The aim of our study was to examine mean platelet volume (MPV), platelet mass (PM) and platelet count (PC), which are practical indicators of platelet activity in preoperative and postoperative periods of the patients with hydatid cyst.

**Methods:**

In this retrospective study we evaluated 72 patients admitted to clinic of chest surgery with a diagnosis of pulmonary hydatid cyst in our hospital between January, 2006, and October, 2008. The MPV, PC, and PM were evaluated by complete blood count. PM was calculated by multiplying MPV and PLT.

**Results:**

Preoperative MPV values of the patients was found to be significantly higher than postoperative MPV values (mean: 8.07±0.83, 7.78±0.87, p= 0.002). Preoperative PM values (median: 2456.75, range: 1013.70–5046.60) was found to be higher when compared to postoperative PM values (median: 2280.80, range: 134.20–4042.60) (p= 0.039). PC values were not significantly different between two periods (Preoperative-postoperative PC mean values: 320.48±98.42, 307.29±96.45, p= 0.286).

**Conclusion:**

In this study, we demonstrated that there were no statistical difference in PC for both periods but, MPV and PM were found statistical higher than postoperative period in preoperative period. PM and PM can be used as markers of disease activity in patients who undergo surgical resection of hydatid cyst.

## Introduction:

*Echinococcosis* is a near-cosmopolitan zoonosis caused by adult or larval stages of tapeworms (cestodes) into the genus *Echinococcus* (family Taeniidae). Larval infection (hydatid disease, hydatidosis) is characterized with long term growth of metacestode (hydatid) cysts in the intermediate host. Hydatid cyst (HC) is a major economic and public health problem in many undeveloped and developing countries.^1^,^2^ Echinococcosis is an increasing public health concern and they can be regarded as emerging or re-emerging diseases.^3^ The liver (75%) and the lungs (15%) are the most common involved sites in HC.^4^ Treatment of HC is based on considerations of the size, location, and manifestations of cysts and the overall health of the patient. Surgery is the principal definitive method and the first choice of the treatment. Since surgery may be complicated due to the possibility of the hydatid cyst rupture. HC surgery should be performed by experienced surgeons.^5^

Platelets contain an array of potent proinflammatory substances, and thus they are regarded as mediator cells in inflammation.^6^ They are potent effector cells in the protection against helminthes.^7^ In the case of helminthic infestations, IgE-dependent killing mechanisms were detected, and it was demonstrated that platelets were capable of killing parasites independent from leukocytes.^8^ Increased mean platelet volume (MPV) is the result of increased proportion of young platelets in the circulation (because platelets decrease in size as they age) and is suggestive of increased platelet production and/or destruction.^9^

Although the measurement of MPV has been available since the 1970’s, its relationship to platelet count and clinical significance of this relation has not been understood in CH. Also specific platelet responses in patients with parasite infestation have not been extensively identified. First objective of the present study was to evaluate MPV, platelet mass (PM) and platelet count (PC), which are practical indicators of platelet activity in preoperative hydatid cyst patients. Our second objective was to investigate whether platelet activity would change in postoperative period in patients with hydatid cyst.

## Material and Methods:

In this retrospective study we evaluated 72 patients admitted to clinic of chest surgery with a diagnosis of pulmonary hydatid cyst in Ataturk Chest Diseases and Thoracic Surgery Training and Research Hospital between January, 2006, and October, 2008. The diagnosis of hydatid cyst made base on clinical findings, serological methods as antibodies anti Echinococcus, and imaging techniques, established with the operation findings. Demographic features, medical history, characteristics of the cysts (size, structure, number of cysts) were recorded for each patient. We did not start to the patients antiparasiter drug in preoperative periods. Hovewer, albendazole was given in postoperative period for a month. We did not determine a control group in the study.

Blood samples were obtained on the first preoperative day and at the 30^th^ day of the postoperative period. The complete blood count (CBC) test was performed on these blood samples. We assessed CBC by an automatic hematologic analyser (Beckman Coulter LH780, USA). 0.5 ml citrat for 2 ml blood sample was applied as an anticoagulant for each blood sample. The MPV, PC, and PM were evaluated. Reference values for our laboratory are 156–373 U 10/L and 6.9–10.8 fL for PC and MPV, respectively. PM was calculated by multiplying MPV and PC.

For statistical analysis, SPSS software (Version 11.5; SPSS Inc, Chicago, Ilionis, USA) applications were used in our study. P value less than 0.05 was considered significant. We used paired sample t test to compare PC with MPV in both preoperative and the postoperative terms. Wilcoxon test was used to compare pre and postoperative PM values. For evaluation of the platelet parameters and the cyst characteristics, independent t test and Mann Whitney U test were used.

## Results:

72 patients were included in the study. The average age of the patients were 35.30 (29.74 for male and 42.30 for female). Thirteen patients (18%) were under 18 year-old at the time of surgery. Four patients had concomitant lung and liver hydatid cyst. Cyst characteristics could not be determined in some patients because of we did not find those in patients files (**Table 1**).

Preoperative MPV (mean: 8.07±0.83) of the patients was found to be significantly higher than postoperative MPV (mean: 7.78±0.87) (p= 0.002). Preoperative PM (median: 2456.75, range: 1013.70–5046.60) was found to be higher on comparing postoperative PM (median: 2280.80, range: 134.20 - 4042.60) (p= 0.039). PC values were not significantly different between two terms (Preoperative PC mean: 320.48±98.42 and postoperative PC mean: 307.29±96.45, p= 0.286) (**Table 2**).

We compared cyst characteristics and platelet parameters in both periods. At the preoperative period we found that MPV was significantly higher in patients who had localized cyst the left lung lobe (mean: 8.23±0.79) than in patients who had localized cyst of the right lung lobe (mean: 7.82±0.84) (p= 0.051). We found no significant difference between platelet parameters (MPV, PC, PM) and cyst characteristics (p>0.05).

## Discussion:

In this study, we found that preoperative and postoperative MPV and PM values of patients with hydatid cyst were significantly different. MPV and PM values of the patients were found to be significantly higher at the preoperative period than the postoperative period. PC values were not significantly different in the periods. MPV was significantly higher in patients who had left lung localized cyst than in patients who had right lung localized cyst. A relationship between the MPV, PM and hydatid cyst has not been studed before.

Platelets have cytotoxic activity in vitro and in vivo against extra-cellular parasites. The toxic process is mediated either by specific antibodies or directly through the parasite itself. The same particular metabolic pathways seem to be implicated in both hemostasis and parasiticidal activity.^10^ Primary hemostasis and cytotoxicity may be regarded as specialized inflammatory responses. Ladhani et al reported that the MPV had been significantly higher in children with malaria compared with those with other medical conditions. Moreover, the MPV values increased as platelet counts fell in both malaria and non-malaria groups.^11^ The increase in MPV values had been reported previously of 26 adults with malaria.^12^ The increase in platelet volume in malaria may be caused by peripheral platelet destruction with early release of large immature platelets from the bone marrow. Wiwanitkit et al reported that a statistically significant decrease of MPV values was detected in the patients with hookworm infection.^13^ No significant difference in platelet count was found between the subjects with or without hookworm infection in the study. Matowicka-Karna J. et al demonstrated that CD62P and CD63 expression increased significantly in patients infected with *E. granulosus*, when compared those to the control group. The analysis of expressions of CD62P and CD63 on blood platelets revealed the presence of intravascular platelet activation.^14^ In another report of the same authors, concentrations of beta-thromboglobulin and platelet factor 4 were found to be higher in patients with echinococcosis.^15^

MPV values increase as platelet count decreases. In this study, we demonstrated that there was no statistical difference in PC values between pre and postoperative terms but MPV and PM values were higher in preoperative term than those in postoperative period. MPV and PM can be useful in follow up of patients with hydatid cyst disease. To the best of our knowledge, our study is the first report that demonstrates this relationship. Limitations of this study include its retrospective design, without any control group and owing to small sample size. Further studies to demonstrate the association between parasitic infestations and platelet parameters should be performed. Other parasitic infections should be included. MPV and PM can be used as markers of disease activity in patients who undergo surgical resection of hydatid cyst.

## Figures and Tables

**Table 1.** Characteristics of hydatid cyst

The number of cysts	Only one cyst in 53 patients, more than one cyst in 11 patients, no data for 8 patients
The size of cysts	Small cyst in 55 patients, giant cyst for 9 patients, no data for 8 patients
Cysts localization	The right lob in 29 patients, the left lobe in 36 patients, bilateral in 6 patients, no data for only one patient
The structure of cysts	Intact in 53 patients, perforated in 14 patients, no data for 5 patients

**Table 2:** The average (mean ± standard deviation), (median, min-max) of platelet parameters of pre- and postoperative in pulmonary cyst hydatid patients.

	**Preoperative**	**Postoperative**	**p**
	**(mean ± std dev)**	**(mean ± std dev)**	
MPV	8.07 ± 0.83	7.78 ± 0.87	0.002
PC	320.48 ± 98.42	307.29 ± 96.45	0.286
	**(median,min-max)**	**(median,min-max)**	
PM	2456.75, 1013.70–5046.60	2280.80, 134.20 - 4042.60	0.039

MPV=Mean platelet volume, PC=Platelet Count, PM=Platelet Mass

## References

[1] McManus DP Zhang W Li J Bartley PB Echinococcosis Lancet 2003 362 1295 304 14575976 10.1016/S0140-6736(03)14573-4

[2] Safioleas M Nikiteas N Stamatakos M Safioleas C Manti CH Revenas C Echinococcal cyst of the subcutaneous tissue: a rare case report Parasitol Int 2008 57 236 8 18203655 10.1016/j.parint.2007.11.002

[3] Moro P Schantz PM Echinococcosis: a review Int J Infect Dis 2009 13 125 33 18938096 10.1016/j.ijid.2008.03.037

[4] Manterola C Benavente F Melo A Vial M Roa JC Description of Echinococcus granulosus genotypes in human hydatidosis in a region of southern Chile Parasitol Int 2008 57 342 6 18434242 10.1016/j.parint.2008.02.005

[5] Ozaras R Aybar Y Kantarci F Mert A Bilir M Hydatid cyst of the lesser sac Intern Med 2007 46 331 2 17380005 10.2169/internalmedicine.46.6288

[6] Klinger MH Platelets and inflammation Anat Embryol (Berl) 1997 196 1 11 9242884 10.1007/s004290050075

[7] Bout D Joseph M Pontet M Vorng H Deslee D Capron A Rat resistance to schistosomiasis: platelet-mediated cytotoxicity induced by C-reactive protein Science 1986 231 153 6 3079916 10.1126/science.3079916

[8] Joseph M Auriault C Capron A Vorng H Viens P A new function for platelets: IgE-dependent killing of schistosomes Nature 1983 303 810 2 6866081 10.1038/303810a0

[9] Catal F Bavbek N Bayrak O Uz E Isik B Karabel M Platelet parameters in children with upper urinary tract infection: is there a specific response? Ren Fail 2008 30 377 81 18569910 10.1080/08860220801947389

[10] Polack B Peyron F Auriault C Platelet cytotoxicity against parasites Nouv Rev Fr Hematol 1991 33 317 22 1780238

[11] Ladhani S Lowe B Cole AO Kowuondo K Newton CR Changes in white blood cells and platelets in children with falciparum malaria: relationship to disease outcome Br J Haematol 2002 119 839 47 12437669 10.1046/j.1365-2141.2002.03904.x

[12] Fajardo LF Rao S Platelet enlargement in malaria Mil Med 1971 136 463 4 5005443

[13] Wiwanitkit V Soogarun S Saksirisampant W Suwansaksri J Platelet parameters in subjects infected with hookworm Platelets 2003 14 391 3 14602553 10.1080/09537100310001612407

[14] Matowicka-Karna J Kemona H Dymicka-Piekarska V Butkiewicz A [The secretory activity of blood platelets--beta-thromboglobulin and platelet factor 4 in echinococcosis] Pol Merkur Lekarski (Abstract) 2005 19 172 4 16245426

[15] Matowicka-Karna J Kemona H Dymicka-Piekarska V Butkiewicz A Activation of blood platelets in echinococcosis--CD62P and CD63 expression Parasitol Res 2006 98 214 7 (Polish) 16333666 10.1007/s00436-005-0038-2

